# Novel approaches leading towards peptide GPCR de‐orphanisation

**DOI:** 10.1111/bph.14950

**Published:** 2020-02-03

**Authors:** Alexander S. Hauser, David E. Gloriam, Hans Bräuner‐Osborne, Simon R. Foster

**Affiliations:** ^1^ Department of Drug Design and Pharmacology University of Copenhagen Copenhagen Denmark; ^2^ Department of Biochemistry and Molecular Biology, Monash Biomedicine Discovery Institute Monash University Clayton VIC Australia

## Abstract

The discovery of novel ligands for orphan GPCRs has profoundly affected our understanding of human biology, opening new opportunities for research, and ultimately for therapeutic development. Accordingly, much effort has been directed towards the remaining orphan receptors, yet the rate of GPCR de‐orphanisation has slowed in recent years. Here, we briefly review contemporary methodologies of de‐orphanisation and then highlight our recent integrated computational and experimental approach for discovery of novel peptide ligands for orphan GPCRs. We identified putative endogenous peptide ligands and found peptide receptor sequence and structural characteristics present in selected orphan receptors. With comprehensive pharmacological screening using three complementary assays, we discovered novel pairings of 17 peptides with five different orphan GPCRs and revealed potential additional ligands for nine peptide GPCRs. These promising findings lay the foundation for future studies on these peptides and receptors to characterise their roles in human physiology and disease.

**Abbreviations**
MS: mass spectrometry

## 1 GPCRS AND DE‐ORPHANISATION

**Figure 1:**
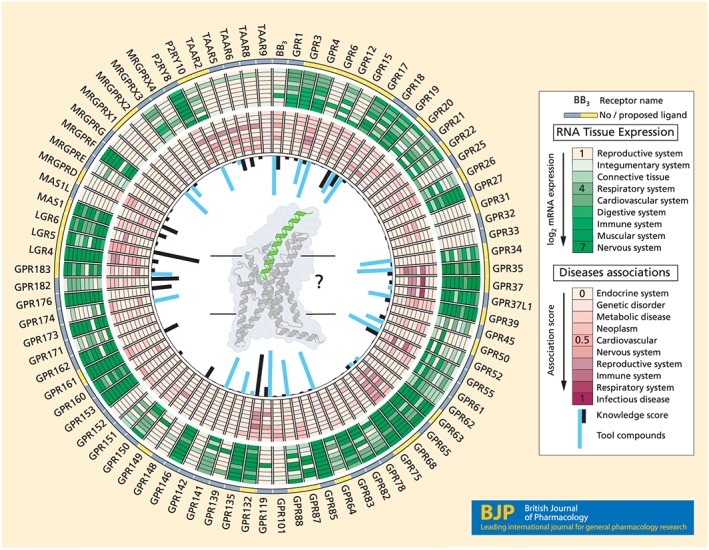
Knowledge state for class A orphan GPCRs. There are 84 class A orphans receptors (excluding tentative pseudogenes), as classified by IUPHAR Committee on Receptor Nomenclature and Drug Classification (NC‐IUPHAR). These receptors generally have low sequence similarity to non‐orphans, making it more challenging to garner reliable data on their evolutionary history or 3D structure than for other GPCRs. Nonetheless, 34 orphan receptors have proposed endogenous ligands (*yellow boxes*), whereas the majority do not (*black boxes*). Gene expression data reveals abundant and ubiquitous tissue expression for many orphan receptors (Lachmann et al., 2018; *green ring*, *darker shading denotes higher abundance*). Aggregated disease associations for orphan receptors from OpenTargets (Carvalho‐Silva et al., 2019) highlight the clinical relevance and therapeutic potential across disease areas (*purple ring*, *darker shading denotes stronger association*). Inner ring: orphan GPCR publication/knowledge scores (*black*) and tool compounds listed on the ChEMBL database (*blue*; Nguyen et al., 2017)

Approximately 30% of the ~400 non‐olfactory human GPCRs have not been definitively paired with endogenous ligands and are designated as “orphan” receptors (Alexander et al., 2019; Laschet, Dupuis, & Hanson, 2018). These receptors represent a wealth of unexplored biology and are likely to be involved in physiological and disease processes (Figure 1). As a recent case‐in‐point, rare‐variant genomic analyses of a large clinical population have recently identified disease associations for numerous orphan GPCRs (Dershem et al., 2019). Equally, as around one third of FDA‐approved drugs mediate their effects via GPCRs (Hauser, Attwood, Rask‐Andersen, Schiöth, & Gloriam, 2017), orphan receptors also hold considerable promise for drug discovery programmes.

GPCR de‐orphanisation—that is, pairing a GPCR with its endogenous ligand(s)—reached its zenith in the late 1990s and early 2000s, with the coalescence of significant investment from the pharmaceutical industry, development of high‐throughput reverse pharmacology approaches, and sequencing of the human genome. This period saw around 10 de‐orphanisations each year, including several success stories that have progressed through drug discovery pipelines to become the targets of approved therapeutic agents, such as neurokinin and orexin receptors (Civelli et al., 2013). However, despite advances in GPCR research, progress in de‐orphanisation has slowed in the intervening years (www.guidetopharmacology.org/latestPairings.jsp; for reviews, see Alexander et al., 2019; Civelli et al., 2013; Laschet et al., 2018). On one hand, this is unsurprising as, inter alia, those targets exhibiting high protein sequence homology with liganded receptors or those responding to known physiological ligands have already been paired. On the other hand, an inherent problem with orphan GPCRs is that their function and signalling pathway(s) are typically unknown. This has necessitated methods utilising chimeric G proteins or β‐arrestin recruitment assays that direct cellular responses to a discrete readout of receptor activation (Ozawa, Lindberg, Roth, & Kroeze, 2010). Given the pleiotropic nature of GPCR signalling, these approaches may have overlooked important receptor–ligand interactions. Indeed, a β‐arrestin recruitment assay screen of ~5,300 candidate endogenous ligands against 82 orphan receptors only identified a single proposed orphan GPCR ligand (Southern et al., 2013). Accordingly, new and different strategies are required to discover the endogenous ligands for the remaining intractable orphan GPCRs.

## 2 APPROACHES FOR PEPTIDE‐GPCR DE‐ORPHANISATION

Peptide ligands and hormones are fundamental physiological mediators that primarily act on GPCRs. Given their involvement in diverse physiological processes, intensive research has been directed towards the identification of peptide receptors and their corresponding endogenous peptide ligands. Indeed, following completion of the human genome, it became clear that the number of peptide receptors exceeded the known peptide ligands (Civelli et al., 2013). Likewise, based on the fraction of peptide‐activated receptors, ~25 https://www.guidetopharmacology.org/GRAC/FamilyDisplayForward?familyId=16 were estimated to have endogenous peptide ligands (Vassilatis et al., 2003). This stimulated renewed experimental and bioinformatic efforts to identify candidate peptide precursors and peptides.

There have been some notable successes, including peptide ligands for https://www.guidetopharmacology.org/GRAC/ObjectDisplayForward?objectId=119 and https://www.guidetopharmacology.org/GRAC/ObjectDisplayForward?objectId=143, which have been implicated in feeding behaviours in mice (Gomes et al., 2013; Gomes et al., 2016). To this end, mass spectrometry (MS) has enabled the discovery of several bioactive peptides (Fricker et al., 2000; Hatcher et al., 2008), even though it is very difficult to detect the inherently limited temporal and spatial expression of secreted peptides in mixed samples containing large quantities of other proteins. MS has been applied to peptide ligand screening, as it is label‐free and unbiased with respect to signalling pathways (Yen et al., 2017). Recently, HPLC and MS of bile and cell culture supernatants have led to the discovery of a post‐translationally modified peptide, S‐geranylgeranyl‐L‐GSH as a potent endogenous ligand for the orphan receptor https://www.guidetopharmacology.org/GRAC/ObjectDisplayForward?objectId=164 (Lu, Wolfreys, Muppidi, Xu, & Cyster, 2019).

The genetic encoding of peptide sequences affords great opportunities for the development of sequence‐based computational methods to identify novel peptides and precursors. These include analyses of shared motifs within precursors (Baggerman, Liu, Wets, & Schoofs, 2005) and the development of probability‐based models using common peptide sequence features (Mirabeau et al., 2007). These computational approaches led to the discovery of spexin and augurin as proposed (although not yet confirmed) endogenous ligands for https://www.guidetopharmacology.org/GRAC/FamilyDisplayForward?familyId=27 and scavenger receptors respectively. These successes notwithstanding, it remains challenging to accurately predict novel peptide ligands using knowledge of existing ligands and sequence data (Ozawa et al., 2010), particularly due to the extensive post‐translational processing of peptides and the complexity of peptide–receptor signalling.

## 3 IDENTIFICATION OF CANDIDATE ENDOGENOUS PEPTIDES AND ORPHAN RECEPTOR TARGETS

**Figure 2:**
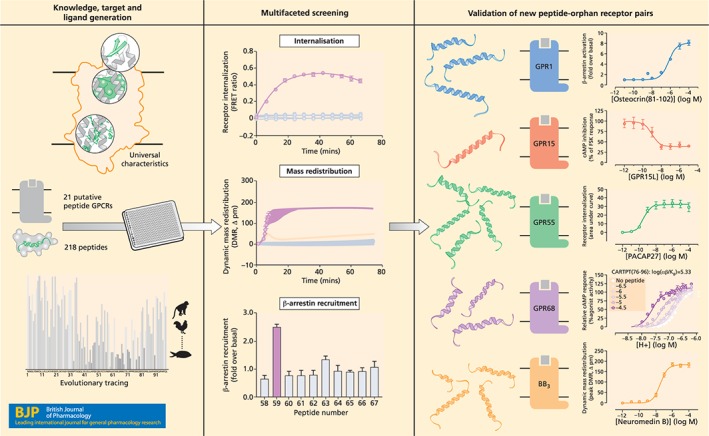
Discovery of novel peptides for orphan GPCRs. Putative peptide orphan receptors were selected based on molecular sequence characteristics (*top left*). An endogenous peptide library was designed from evolutionary tracing and putative cleavage sites found within potential precursor proteins (bottom left); 218 peptides were screened against 21 orphan GPCRs in three independent functional assays covering multiple signalling pathways (*middle*). Five orphan GPCRs (GPR1, GPR15, GPR55, GPR68, and BB_3_) were paired with 17 peptides and validated in at least two orthogonal assays (examples on the right). These novel peptide–receptor interactions represent unexplored aspects of human physiology with considerable implications for drug discovery efforts

We have recently embarked on a large combined computational and experimental approach to identify orphan GPCR ligands (Figure 2; Foster et al., 2019). Predicated on the hypothesis that there are undiscovered endogenously produced peptide ligands for orphan receptors, we began by collecting all publicly available information on known endogenous receptor–ligand pairings. Our quantitative analyses demonstrated that endogenous peptide ligands far outnumber endogenous small molecule ligands, are larger, and often have higher affinity to their cognate receptors. Evolutionary analyses across hundreds of eukaryotic organisms revealed that co‐evolution of peptide ligand–receptor pairs has made peptides the most adaptive, widely utilised, and versatile type of human signalling molecules.

Building on these observations, we identified defining sequence and structural characteristics for peptide ligands and receptors and then leveraged these to mine the human proteome for potential peptide ligands and predicted putative peptide‐binding receptors. In brief, we queried the proteome for new peptide ligand precursors based on secretion motifs and combined this with evolutionary conservation analyses of all known peptide ligands and their precursors. This revealed that peptide‐coding regions are considerably more conserved than other parts of the precursor. Hence, using a machine‐learning model and prioritising the most conserved regions of each precursor candidate between conserved dibasic cleavage motifs, we generated a library of putative endogenous peptide ligands for experimental testing. The final library comprised 218 custom‐synthesised peptides, including 49 known peptide ligands for class A GPCRs. In parallel, based on molecular sequence signatures of known peptide receptors, we predicted the class A orphan receptors most likely to be activated by peptides, and selected 21 for further characterisation.

To maximise the likelihood of capturing peptide‐dependent orphan GPCR activation, regardless of signalling pathway, we evaluated our putative endogenous peptide ligands in three parallel assay platforms: dynamic mass redistribution (Schröder et al., 2011), real‐time receptor internalisation (Foster & Bräuner‐Osborne, 2018), and β‐arrestin recruitment (PRESTO‐Tango; Kroeze et al., 2015). Each of these assays has strengths and limitations individually: Dynamic mass redistribution assays detect G protein‐mediated responses from endogenously expressed proteins, as well as overexpressed receptors, but do not directly measure β‐arrestin signalling (Grundmann et al., 2018). The internalisation assay can detect β‐arrestin‐dependent and independent trafficking but relies on an N‐terminal SNAP tag which could potentially modulate ligand binding. The Tango assay is a sensitive downstream genetic readout for β‐arrestin recruitment, although the signal amplitude varies between receptors and it does not report activation for all GPCRs (Kroeze et al., 2015). For logistical reasons, our screens were performed in recombinant expression systems (e.g., modified HEK cells), and it is conceivable that these could lack required signalling partners for orphan receptors. However, in combination, these assays provide complementary coverage of GPCR‐mediated signalling and overcome significant limitations of previous de‐orphanisation efforts.

## 4 NEW PEPTIDE LIGANDS FOR ORPHAN GPCRS

Using our multifaceted experimental approach, we paired five “orphan” receptors with 17 peptides that represent potential novel endogenous ligands (Foster et al., 2019) (Table 1). These include peptides for https://www.guidetopharmacology.org/GRAC/ObjectDisplayForward?objectId=82, https://www.guidetopharmacology.org/GRAC/ObjectDisplayForward?objectId=87, https://www.guidetopharmacology.org/GRAC/ObjectDisplayForward?objectId=109, https://www.guidetopharmacology.org/GRAC/ObjectDisplayForward?objectId=114, and https://www.guidetopharmacology.org/GRAC/ObjectDisplayForward?objectId=40 receptors, validated in at least two orthogonal assays (discussed below). We also identified indicative pairings for five other orphan receptors using the β‐arrestin recruitment assay and potential secondary peptide ligands for nine known peptide GPCRs. Conversely, we identified nine peptides that elicited clear responses in background cells, which could be considered as “orphan peptides” without a currently known endogenous GPCR or non‐GPCR target.

**Table 1:** New proposed peptide ligands for orphan GPCRs

GPCR	Precursor/protein name	Peptide position within precursor	Amino acid sequence	Peptide novelty	Signalling assays/endpoint	pEC_50_	Comment
GPR1	Osteocrin	OSTN (81–102)	KRSFSGFGSPLDRLSAGSVDHK	Variant	β‐arrestin recruitment (TANGO and DiscoverX)	5.6–6.2	—
GPR1	Gastrin‐releasing peptide	GRP (24–50)	VPLPAGGGTVLTKMYPRGNHWAVGHLM	Known (GRP)	β‐arrestin recruitment (TANGO and DiscoverX)	5.3–6.4	BB_1_ and BB_2_ receptor agonists
GPR1	Cholecystokinin	CCK (71–103)	KAPSGRMSIVKNLQNLDPSHRISDRDYMGWMDF	Known (CCK‐33)	β‐arrestin recruitment (TANGO and DiscoverX)	5.3–8.0	CCK_1_ and CCK_2_ receptor agonists
GPR15	Protein GPR15L	GPR15L (71–81)	LWVVPGALPQV	Variant	DMR, internalisation, cAMP, β‐arrestin recruitment (TANGO)	5.0–6.8	C‐terminal truncation of GPR15L (Suply et al., 2017)
GPR55	MANSC domain‐containing protein 1	MANSC1 (415–431)	KRYSRLDYLINGIYVDI	New	DMR, internalisation	4.9–5.5	Response >1 μM
GPR55	Pituitary adenylate cyclase‐activating polypeptide	ADCYAP1 (132–158)	HSDGIFTDSYSRYRKQMAVKKYLAAVL	Known (PACAP‐27)	DMR, internalisation	9.5–10.3	PAC_1_, VPAC_1_, and VPAC_2_ receptor agonists
GPR55	Sperm‐associated antigen 11B	SPAG11B (61–103)	DLLPPRTPPYQVHISHREARGPSFRICVDFLGPRWARGCSTGN	New	DMR, internalisation	4.8–5.7	Response >1 μM
GPR55	Secretogranin‐1	CHGB (511–532)	KRLGALFNPYFDPLQWKNSDFE	New	DMR, internalisation	5.3–5.6	—
GPR55	β‐microseminoprotein	MSMB (91–114)	EDCKYIVVEKKDPKKTCSVSEWII	New	DMR, internalisation	5.6–6.7	—
GPR55	Clusterin‐like protein 1	CLUL1 (52–77)	ALTGIKQMKIMMERKEKEHTNLMSTL	New	DMR, internalisation	6.3–6.7	—
GPR68	Osteocrin	OSTN (115–133)	RFGIPMDRIGRNRLSNSRG	Variant	DMR, internalisation, Ca^2+^, cAMP	5.3–6.4	Positive allosteric modulator of proton response at GPR68
GPR68	Cocaine‐ and amphetamine‐regulated protein	CARTPT (76–96)	YGQVPMCDAGEQCAVRKGARI	Variant	DMR, internalisation, Ca^2+^, cAMP	5.3–6.0	Positive allosteric modulator of proton response at GPR68
GPR68	Pro‐opiomelanocortin	PENK (140–162)	RRPVKVYPNVAENESAEAFPLEF	Variant	DMR, internalisation, Ca^2+^, cAMP	5.1–5.9	Positive allosteric modulator of proton response at GPR68
BB_3_	Neuromedin B	NMB (47–56)	GNLWATGHFM	Known (neuromedin B)	DMR, internalisation, IP_1_, β‐arrestin recruitment (TANGO)	5.7–7.4	BB_1_ and BB_2_ receptor agonists
BB_3_	Neuromedin‐U	NMU (104–114)	FLFHYSKTQKL	Variant	DMR, internalisation, IP_1_	5.6–5.7	—
BB_3_	Proenkephalin‐A	PENK (210–234)	YGGFMRRVGRPEWWMDYQKRYGGFL	Known (Peptide E)	DMR, internalisation, IP_1_, β‐arrestin recruitment (TANGO)	5.2–5.4	Response >1 μM
BB_3_	Gastrin‐releasing peptide	GRP (24–50)	VPLPAGGGTVLTKMYPRGNHWAVGHLM	Known (GRP)	DMR, internalisation, IP_1_, β‐arrestin recruitment (TANGO)	5.7–6.4	BB_1_ and BB_2_ receptor agonists

Abbreviations: DMR, dynamic mass redistribution (Corning EPIC); internalisation, SNAP‐tag based real‐time internalisation (Cisbio); β‐arrestin recruitment assays (TANGO from Kroeze et al., 2015, and DiscoverX); IP_1_, inositol monophosphate accumulation (Cisbio); cAMP, cAMP accumulation (Cisbio) and GloSensor assays (Promega).

### 4.1 GPR1

We discovered three peptides that robustly activated GPR1 (recently renamed chemerin receptor 2; Kennedy & Davenport, 2018). These include a new peptide derived from the osteocrin precursor and two known peptides https://www.guidetopharmacology.org/GRAC/LigandDisplayForward?ligandId=612 and https://www.guidetopharmacology.org/GRAC/LigandDisplayForward?ligandId=860. Consistent with published reports (Alexander et al., 2019), these responses were confirmed in two different β‐arrestin recruitment assays, while no G protein signalling was observed. These findings suggest that GPR1 is a β‐arrestin biased receptor, which will be of interest to clarify in future studies, particularly given the broad expression profile and pathophysiological implications for GPR1/chemerin2 (Kennedy & Davenport, 2018).

### 4.2 GPR15

We identified a novel 11‐amino acid peptide derived from an uncharacterised gene *C10orf99* as a GPR15 ligand. We then investigated longer peptide variants and identified a 57‐residue peptide as the most potent GPR15 ligand. During the course of our project, this same pairing was independently reported by Novartis and confirmed by another research group (Ocon et al., 2017; Suply et al., 2017), and this ligand has since been renamed as GPR15L. Nonetheless, whereas Suply et al. (2017) isolated GPR15L from pig colon, we used an entirely different computational approach that discovered additional peptide cleavage variants demonstrating the importance of the carboxy‐terminus (Foster et al., 2019). The GPR15 and GPR15L signalling axis is an emerging therapeutic target for colon and skin inflammation (Suply et al., 2017).

### 4.3 GPR55

We identified five novel peptides and https://www.guidetopharmacology.org/GRAC/LigandDisplayForward?ligandId=2257 as GPR55 ligands using unbiased mass redistribution and internalisation assays. Intriguingly, PACAP‐27 (a known class B receptor ligand) activated GPR55 with comparable picomolar potency to its cognate receptor https://www.guidetopharmacology.org/GRAC/ObjectDisplayForward?objectId=370 (Alexander et al., 2019). GPR55 preferentially couples to G_12/13_ and is a challenging receptor target, so further studies are required in G protein assays and relevant physiological contexts. Equally, the potential interaction of GPR55 with PAC_1_ is worthy of investigation, particularly given the recent description of crosstalk between the https://www.guidetopharmacology.org/GRAC/ObjectDisplayForward?objectId=319 and the orphan receptor https://www.guidetopharmacology.org/GRAC/ObjectDisplayForward?objectId=130 (Wang et al., 2019).

### 4.4 GPR68

GPR68 is a proton‐sensing GPCR that is currently attracting interest as a potential target for airway inflammation, CNS disorders, and cancer (Huang et al., 2015). Nonetheless, we observed that GPR68 displays many characteristics of peptide‐activated GPCRs (Foster et al., 2019), and we discovered multiple peptides that potentiate the proton‐mediated GPR68 signalling. These include undescribed peptide variants from osteocrin and cocaine‐ and amphetamine‐regulated transcript protein precursors. These ligands represent the first peptide positive allosteric modulators of GPR68, with approximately twofold improved allosteric activity (log (ab/K_B_)) over the small molecule compound https://www.guidetopharmacology.org/GRAC/LigandDisplayForward?ligandId=9155 (Huang et al., 2015).

### 4.5 BB_3_

The bombesin family receptor BB_3_ is weakly activated by bombesin‐like peptides and has been previously described as a “reluctant de‐orphanisation” (Civelli et al., 2013). In our study, we identified https://www.guidetopharmacology.org/GRAC/LigandDisplayForward?ligandId=613 and gastrin‐releasing peptide‐dependent BB_3_ activation at high nanomolar concentrations, more potent than previously reported, but still lower potency than for the BB_1_ receptor. As receptor knockout mice develop mild obesity, BB_3_ receptors have been implicated in feeding behaviour regulation, potentially in concert with other bombesin receptors (Civelli et al., 2013). Interestingly, due to its constitutive activity, BB_3_ receptors have recently been suggested to lack an endogenous ligand (Tang et al., 2019).

Collectively, our study has yielded new insights into human peptidergic receptor signalling and revealed several novel putative endogenous peptide–receptor interactions. These pairings require additional research to determine their physiological relevance including, ultimately, supporting in vivo studies. As many orphan receptors were activated with low potency, these may be considered as lead peptides for future studies, as the precise physiologically relevant cleavage variant and post‐translational modifications for these peptides remain to be identified. We would therefore encourage further characterisation of our proposed peptide–receptor pairings, in particular by testing peptide variants in relevant biological systems with endogenous receptor expression.

Our identification of new peptide–receptor pairings strongly validates our combinatorial computational and experimental approach for GPCR de‐orphanisation. Nonetheless, in light of the vast number of potential peptides encoded in the human proteome and the permutations of post‐translational modifications, it is possible that the optimal peptide ligands (or peptide‐activated receptors) were not tested. Moreover, we could not account for peptide cleavage and truncation, for example, by plasmin and https://www.guidetopharmacology.org/GRAC/ObjectDisplayForward?objectId=1612, which is important for other endogenous neuropeptide and chemokine ligands (Richter et al., 2009; Torang et al., 2016). Interestingly, new large‐scale transcriptome and proteome studies now have improved coverage of human peptides and proteins, which may also lead to the discovery of previously unappreciated protein products (Jiang et al., 2019). Orphan receptors may also require additional signalling partners that were absent from our experimental setups, such as other GPCRs or receptor activity‐modifying proteins (Lorenzen et al., 2019; Wang et al., 2019). Alternatively, these receptors may not have peptide ligands or be constitutively active (Martin, Steurer, & Aronstam, 2015), or their activating molecules may be produced exogenously, as suggested for microbiome‐derived ligands for https://www.guidetopharmacology.org/GRAC/ObjectDisplayForward?objectId=126 (Cohen et al., 2017). These are all potential areas for future investigation.

## 5 CONCLUDING REMARKS

The discovery of new orphan GPCR ligands regularly has substantial impact, and each of our peptide*–*receptor pairings opens up new avenues of research. Indeed, all paired receptors and the majority of their peptide ligands have been implicated in disease, suggesting high translational potential to druggable targets and ligands. Hence, our new approach and findings will have broad appeal and effects across research fields and therapeutic areas.

### 5.1 Nomenclature of targets and ligands

Key protein targets and ligands in this article are hyperlinked to corresponding entries in http://www.guidetopharmacology.org, the common portal for data from the IUPHAR/BPS Guide to PHARMACOLOGY (Harding et al., 2018), and are permanently archived in the Concise Guide to PHARMACOLOGY 2019/20 (Alexander et al., 2019).

## CONFLICT OF INTEREST

The authors declare no conflicts of interest.
